# Wild *Cicer* species exhibit superior leaf photosynthetic phosphorus‐ and water‐use efficiencies compared with cultivated chickpea under low‐phosphorus conditions

**DOI:** 10.1111/nph.70185

**Published:** 2025-05-05

**Authors:** Jiayin Pang, Simiao Li, Ulrike Mathesius, Jens Berger, Weina Zhang, Komal D. Sawant, Rajeev K. Varshney, Kadambot H. M. Siddique, Hans Lambers

**Affiliations:** ^1^ School of Biological Sciences The University of Western Australia Perth WA 6001 Australia; ^2^ The UWA Institute of Agriculture The University of Western Australia Perth WA 6001 Australia; ^3^ State Key Laboratory of Grassland Agro‐ecosystems, Key Laboratory of Grassland Livestock Industry Innovation, Ministry of Agriculture and Rural Affairs, College of Pastoral Agriculture Science and Technology Lanzhou University Lanzhou 730000 China; ^4^ Division of Plant Sciences, Research School of Biology Australian National University Canberra ACT 2601 Australia; ^5^ Agriculture and Food CSIRO Floreat WA 6010 Australia; ^6^ School of Biological and Food Processing Engineering Huanghuai University Zhumadian 463000 China; ^7^ Department of Botany Nowrosjee Wadia College Pune Maharashtra 411001 India; ^8^ Centre for Crop and Food Innovation, WA State Agricultural Biotechnology Centre Food Futures Institute, Murdoch University Murdoch WA 6150 Australia

**Keywords:** *Cicer echinospermum*, *Cicer reticulatum*, domestication, intercellular CO_2_, leaf mass per area, leaf nitrogen concentration, photosynthesis, photosynthetic nitrogen‐use efficiency

## Abstract

- Domesticated chickpea cultivars exhibit limited genetic diversity. This study evaluated the effects of chickpea domestication on phosphorus (P)‐use efficiency (PUE) under low‐P conditions, using a diverse *Cicer* collection, including wild species.
- Two wild *Cicer* species – 54 *C. reticulatum* accessions and 15 *C. echinospermum* accessions, and seven domesticated *C. arietinum* accessions were grown in low‐P soil.
- All three species exhibited significant variation in physiological PUE, leaf gas exchange characteristics, photosynthetic PUE (PPUE), and photosynthetic N‐use efficiency (PNUE), with greater variation in wild *Cicer* species than in domesticated *C. arietinum*. Domestication increased shoot growth and total leaf area but reduced root mass ratio. Compared with domesticated *C. arietinum*, wild *Cicer* species had lower stomatal conductance and higher leaf mass per area, associated with lower intercellular CO_2_ concentrations and higher water‐use efficiency (WUE). Elevated leaf nitrogen concentrations in wild *Cicer* were likely associated with enhanced photosynthetic capacity, partially compensating for reduced stomatal conductance.
- Wild *Cicer* species demonstrated higher PPUE but lower PNUE than domesticated chickpea, with increased WUE exhibiting a trade‐off with PNUE. These findings highlight the potential of wild *Cicer* species as valuable genetic resources for enhancing PPUE in chickpea improvement programmes.

## 摘要

- 本研究针对栽培型鹰嘴豆遗传多样性较为受限的现状，通过整合*Cicer*属野生种质资源，系统评估了驯化过程对鹰嘴豆在低磷条件下磷利用效率的影响机制。
- 在低磷条件下种植了76份材料，涵盖两个野生鹰嘴豆物种(包括54份 *C. reticulatum*材料和15 份*C. echinospermum*材料)，以及一个栽培种(*C. arietinum*，共7份材料)。
- 结果表明:(1)三个鹰嘴豆物种在生理磷利用效率、叶片气体交换特性、光合磷利用效率和光合氮利用效率等方面均表现出显著的种内变异，且野生种的变异幅度大于栽培种。(2)驯化过程中虽然提高了地上部生物量和总叶面积，但降低了根质量比。(3)相比于栽培种，野生种叶片的气孔导度较低、比叶重较高，并表现出较低的叶片胞间二氧化碳浓度和更高的水分利用效率。(4)野生种叶片中较高的氮浓度可能有助于增强其光合能力，在一定程度上弥补了气孔导度降低所带来的不利影响。
- 与栽培种相比，野生种鹰嘴豆具有更高的光合磷利用效率，但光合氮利用效率较低，且水分利用效率的提升与光合氮利用效率之间存在权衡关系。该研究结果显示，野生鹰嘴豆在提高光合磷利用效率方面具有巨大潜力，为鹰嘴豆的遗传改良提供了宝贵的遗传资源。

## Introduction

Chickpea (*Cicer arietinum*) is a vital legume crop for food and feed in developing countries (Foyer *et al*., 2016). However, domestication has significantly narrowed its genetic diversity (Abbo *et al*., 2003; Varshney *et al*., 2013, 2021; Marques *et al*., 2020; Khan *et al*., 2024). To address this limitation, the International Crops Research Institute for the Semi‐Arid Tropics (ICRISAT) developed a chickpea reference set of 300 accessions from 29 countries, representing diverse genetic backgrounds (Upadhyaya *et al*., 2008). Studies on this set revealed significant genotypic variation in plant growth, shoot phosphorus (P) content, physiological P‐use efficiency (PUE), leaf photosynthetic characteristics, photosynthetic PUE (PPUE), root morphology, carboxylate exudation, and arbuscular mycorrhizal fungal colonisation (Pang *et al*., 2018a,b, 2023; Wen *et al*., 2020, 2022). However, this reference set included only seven wild *Cicer* accessions due to the limited global collections available at the time (Berger *et al*., 2003; Coyne *et al*., 2020). Wild species are typically genetically much more diverse than their domesticated counterparts due to the domestication bottleneck (Tanksley & McCouch, 1997; Purugganan & Fuller, 2009). While crops are weak competitors in managed systems that minimize stress, wild progenitors such as wild *Cicer* thrive in unregulated low‐fertility environments (Renzi *et al*., 2022). Therefore, screening wild germplasm for nutrient acquisition capacity is crucial. Recent international collaborations have expanded wild *Cicer* collections (von Wettberg *et al*., 2018), enabling exploration of their genetic diversity for abiotic stress tolerance, including low P availability.

*Cicer reticulatum*, the primary gene pool of cultivated chickpea, is fully compatible with domesticated *C. arietinum* (Coyne *et al*., 2020). The secondary gene pool, *C. echinospermum*, exhibits variable compatibility with cultivated chickpea depending on the population (Kahraman *et al*., 2017). Both wild species are restricted to south‐eastern Anatolia, Türkiye, where they inhabit distinct ecological niches shaped by diverse soil substrates and a steep elevational gradient exceeding 1000 m (von Wettberg *et al*., 2018). Therefore, localized adaptation underscores their potential for broadening the genetic base of chickpea and introducing adaptive traits lost during domestication (Croser *et al*., 2003; von Wettberg *et al*., 2018).

Recent studies have explored wild *Cicer* species for genotypic variation under abiotic stress, such as heat (von Wettberg *et al*., 2018), drought (von Wettberg *et al*., 2018; Berger *et al*., 2020), nitrogen (N) deficiency (Marques *et al*., 2020), and aluminium toxicity (Vance *et al*., 2021), biotic stress like *Helicoverpa armigera* infestation (von Wettberg *et al*., 2018), and seed quality traits, including nutrient composition (von Wettberg *et al*., 2018; Sharma *et al*., 2021) and seed size (von Wettberg *et al*., 2018). Given that wild *Cicer* species naturally inhabit low‐P soils, they may possess traits for greater P‐use efficiency.

Phosphorus availability often limits crop production, with up to 80% of applied P fertilizer rendered unavailable to plants due to its conversion into insoluble complexes in acidic and alkaline soils (Raghothama, 1999; Lambers, 2022). Enhancing PUE, defined as increased yield per unit P applied, is an environmentally sustainable strategy (Rose *et al*., 2013). PPUE, the photosynthesis rate per unit of leaf P, is a critical component of PUE, given that photosynthesis contributes over 90% of crop biomass (Veneklaas *et al*., 2012; Cong *et al*., 2020). Despite its importance, few studies have examined the effects of domestication on PUE, particularly PPUE in crops, including the chickpea breeding programme (Chen *et al*., 2024; Wang *et al*., 2024). Previous research demonstrated significant genotypic variation in PUE, including P‐acquisition efficiency, physiological PUE, and PPUE among domesticated *C. arietinum* accessions under low‐P conditions (Pang *et al*., 2018b) and revealed biochemical mechanisms underpinning a high PPUE, such as a different allocation to foliar P fractions, with a lower allocation to Pi and metabolite P but a greater allocation to nucleic acid P (Wen *et al*., 2022). However, the genetic diversity of P acquisition and PPUE in the genus *Cicer* remains unexamined, particularly among wild *Cicer* accessions.

Domestication effects on leaf gas exchange traits vary among crops. For instance, Milla & Matesanz (2017) reported reduced photosynthesis rates during the domestication of *Beta vulgaris*, *Helianthus annuus*, *Solanum lycopersicum*, and *Zea mays*. Conversely, studies have shown an increased photosynthesis rate in domesticated crops compared with their wild relatives, such as chickpea under both N‐deficient and N‐sufficient conditions (Marques *et al*., 2020), cotton (*Gossypium hirsutum*) (Z. Y. Lei *et al*., 2023), and soybean (*Glycine max*) (Chen *et al*., 2024; Pelech *et al*., 2025). Recently, Lei *et al*. (2024) reported stage‐specific increases in photosynthesis rate during domestication, influenced by variation in light intensity. The transition from wild to semiwild accessions resulted in a faster photosynthesis rate and lower leaf mass per area (LMA) under high light intensity, whereas the transition from semiwild to domesticated accessions enhanced photosynthesis under low light intensity. Whether domestication similarly affects leaf gas exchange in *Cicer* grown under sufficient light intensity, particularly under low‐P conditions, is unknown.

Wild species and their relatives generally exhibit thicker leaves and higher LMA than their domesticated counterparts, as observed in *Cicer* (Marques *et al*., 2020) and *Gossypium* (Lei *et al*., 2021; Z. Y. Lei *et al*., 2023). Higher LMA is often associated with resilience to drought by reducing wilting risk (Wright *et al*., 2001). However, a trade‐off between water‐use efficiency (WUE) and photosynthetic N‐use efficiency (PNUE) has been documented in noncrop plants (Field *et al*., 1983; Wright *et al*., 2001; Zhou *et al*., 2024) as well as in *Triticum aestivum* (Van Den Boogaard *et al*., 1997). For example, dry‐site tree species maintain area‐based photosynthesis rates with lower stomatal conductance by increasing leaf N concentrations, thus minimising water loss (Wright *et al*., 2001). Whether this trade‐off exists among *Cicer* species, particularly under low‐P conditions, remains unknown.

In this study, we used the wild *Cicer* collection to evaluate vegetative growth, photosynthesis characteristics, PPUE, and PNUE under low‐P conditions. We hypothesized that: (1) significant variation in leaf gas exchange and PUE exists among wild *Cicer* accessions due to their distinct natural habitats; (2) variation among wild *Cicer* accessions exceeds that in cultivated *C. arietinum*; (3) wild *Cicer* species exhibit greater PPUE than domesticated chickpea; and (4) wild *Cicer* species display a trade‐off between higher instantaneous intrinsic WUE and lower PNUE.

## Materials and Methods

### Plant material and growth conditions

This study involved 69 wild *Cicer* accessions from two wild species: 54 *C. reticulatum* L. accessions and 15 *C. echinospermum* P.H.Davis accessions. Additionally, 7 *C. arietinum* L. accessions (desi types) were included, comprising two Australian commercial cultivars (PBA Drummond and PBA Captain) and five accessions with contrasting traits, such as variation in leaf gas exchange, root morphology, root mass ratio, and carboxylate exudation (Pang *et al*., 2018a,b). Given its narrower natural habitat range and lower genetic variation, reflected in the reduced number of polymorphic loci (88 976) compared with *C. reticulatum* (136 638), as reported by von Wettberg *et al*. (2018), fewer *C. echinospermum* accessions were included in this study. All wild *Cicer* seeds were originally collected from Türkiye in 2013, with details on soil, geographic, and climatic characteristics described by von Wettberg *et al*. (2018). The Australian Grains Genebank (Horsham, Vic., Australia) supplied seeds after multiplication. Table 1 provides the collection sites/country of origin and accession details for wild *Cicer* and cultivated *C. arietinum*.

**Table 1:** List of wild *Cicer* accessions (54 *C. reticulatum* and 15 *C. echinospermum*) and their collection sites in Türkiye, together with seven cultivated *C. arietinum* accessions and their country of origin.

Accession name	Collection site	Accession name	Collection site
***C. reticulatum*** (54)		***C. reticulatum***	
Olgun_026	Ayvalik	Savur_063	Savur
Bari1_062	Baristepe 1	Konak_049	Savur
Bari1_092	Baristepe 1	Savur_036	Savur
Bari2_062	Baristepe 2	Sirna_060	Sirnak
Bari2_072	Baristepe 2	Sirna_063	Sirnak
Bari3_072C	Baristepe 3	Sirna_032	Sirnak
Bari3_100	Baristepe 3	Sirna_036	Sirnak
Besev_075	Beslever	Tasdi_025	Tasdibek
Besev_079	Beslever	Umurl_500	Umurlu
CudiB_005	Cudi	Yanıl_013	Yanilmaz
CudiB_022C	Cudi	Yesil_018	Yesilkoy
CudiA_128	Cudi 2	Yolag_066	Yolagzi
CudiA_152	Cudi 2		
Golko_001	Cukur		
Derei_070	Dereici		
Derei_072	Dereici	***C. echinospermum*** (15)	
Dogan_037	Doganca	Cermi_071	Cermik
Dogan_040	Doganca	Cermi_075	Cermik
Egil‐_065	Egil	Deste_064	Destek
Egil‐_073	Egil	Deste_080	Destek
Ekind_045	Ekinduzu	Gunas_062	Gunasan
Ekind_060	Ekinduzu	Gunas_100	Gunasan
Erenk_001	Erenkaya	Karab_063	Karabahce
Erenk_002	Erenkaya	Karab_172	Karabahce
Golge_031	Golgelikonak	Isoha_003	Kargali
Golge_036	Golgelikonak	Isoha_040	Kargali
Hisar_018	Hisarkaya	Ortan_066	Ortanca
Hisar_025	Hisarkaya	Otlu_004	Otlu
Kalka_064	Kalkan	Otlu_015	Otlu
Kalka_067	Kalkan	S2Drd_061	Siv‐Diyar
Kalka_074	Kalkan	S2Drd_105	Siv‐Diyar
Kayat_065	Kayatepe		
Kayma_001	Kaymakam Ceşmesi		
Kayma_044	Kaymakam Ceşmesi		
Kesen_072	Kesentas	***C. arietinum*** (7)	**Country of origin**
Kesen_075	Kesentas	PBA Drummond	Australia
Oyali_084	Oyali	PBA Captain	Australia
Oyali_112	Oyali	ICC4814	Iran
Pınar_044	Pinardere	ICC2884	Iran
Pınar_056	Pinardere	ICC4418	Iran
Sarik_067	Sarikaya	ICC456	India
Sarik_073	Sarikaya	ICC14799	India

Seeds were sown in plastic pots (8.5 × 8.5 × 18 cm) containing 1.2 kg of a 1 : 9 (w/w) mixture of air‐dried field soil (0–15 cm) and washed river sand. The field soil, collected from Cunderdin Agriculture College, Western Australia (31.64° S, 117.24° E), was a reddish‐brown sandy clay loam (9% silt, 27% clay, 64% sand) (Pang *et al*., 2017). The soil mixture was sieved through a 2‐mm mesh before being analysed by the CSBP Future Analytical Laboratories (Bibra Lake, Australia). The mixture contained 0.2 μg g^−1^ nitrate N, 0.2 μg g^−1^ ammonium N, 4.1 μg g^−1^ Colwell P, 26.4 μg g^−1^ total P, 47.1 μg g^−1^ Colwell K, 8.7 mg g^−1^ organic carbon, with a pH (CaCl_2_) of 7.1 and a P retention index of 9.5. The water content of the soil mixture at 100% pot capacity was 12% (w/w), which was determined before the experiment as described in Pang *et al*. (2017). The soil mixture was watered to 80% pot capacity with a basal nutrient solution containing 15 μg N g^−1^ soil as Ca(NO_3_)_2_.4H_2_O, 3.75 μg N g^−1^ soil as NH_4_Cl, 50 μg S g^−1^ soil as K_2_SO_4_, 4 μg Mn g^−1^ soil as MnSO_4_.H_2_O, 2 μg Zn g^−1^ soil as ZnSO_4_.7H_2_O, 0.5 μg Cu g^−1^ soil as CuSO_4_.5H_2_O, 0.4 μg Mo g^−1^ soil as Na_2_MoO_4_.2H_2_O and 5 μg Fe g^−1^ soil as FeNaEDTA. No extra P was applied to maintain low‐P conditions.

Seeds were treated with P Pickel‐T® (360 g l^−1^ thiram, 200 g l^−1^ thiabendazole) and sown at a depth of 25 mm. Four seeds were planted per pot, with four replicates per accession. The pots were kept in the dark in a temperature‐controlled growth room (4°C) for 3 wk to ensure vernalisation. During this period, plastic sheets covered the tops of the pots to minimize evaporation, and no additional water was added. Radicle emergence (*c*. 2–3 cm long) was observed in some extra pots, but no shoots had emerged by the end of vernalisation.

After vernalisation, the plastic covers were removed, and pots were transferred to a temperature‐controlled glasshouse at The University of Western Australia, Perth, Australia (31.57°S, 115.47°E), with a day temperature of 23°C and a night temperature of 13°C, and 65% relative humidity. Each pot received *c*. 1 g of peat‐based Group N rhizobium (New Edge Microbials, Albury, NSW, Australia) as a water slurry. At 10 d post‐transfer, seedlings were thinned to one per pot, and a 10‐mm plastic bead layer was added to the soil surface to minimize soil evaporation. The plants were grown under natural light for 63 d (June to August) when all accessions were at their vegetative stage. This was to minimize the influence of developmental stage on plant traits and to ensure consistency and comparability across *Cicer* species.

### Plant measurements

Before the final harvest, plant height and branch number were recorded for all plants at their vegetative stage. The branch count included the main stem and primary and secondary branches (≥ 20 mm long). Gas exchange measurements were conducted on young, fully expanded leaves on primary branches between 10:00 h and 12:30 h WST using a LI‐COR 6400 portable photosynthesis system with a red/blue LED light source (LI‐COR, Lincoln, NE, USA). Measurement parameters were set to 1500 μmol m^−2^ s^−1^ photosynthetic photon flux density at the leaf surface, 25°C block temperature, 500 μmol s^−1^ flow rate, and 400 μmol mol^−1^ ambient CO_2_ concentration for the incoming gas stream (Pang *et al*., 2018b). Leaf intercellular CO_2_ concentration (C_i_) was calculated from these measurements. The measured leaflets were collected and scanned at 200 dpi using an Epson V850 scanner (Epson America, Long Beach, CA, USA), and the projected leaf area was analysed using WinRhizo 2009 software (Regent Instruments, QC, Canada). Leaf photosynthesis rate, stomatal conductance, and transpiration rate were expressed based on the actual leaf area in the measuring chamber. The leaves used for gas exchange measurements and one adjacent leaf below (young fully expanded leaves) were collected, dried, and analysed to determine leaf P concentration ([P]), subsequently used to calculate PPUE. The remaining leaves were harvested to determine leaf area as above, and LMA was calculated as the ratio of leaf dry weight to leaf area. Mass‐based photosynthesis rate (*A*_mass_) was derived from the area‐based photosynthesis rate (*A*_area_) divided by LMA. Instantaneous intrinsic WUE at the leaf level was calculated as the ratio of photosynthesis rate to stomatal conductance (Osmond *et al*., 1980).

Roots were separated from the shoots and washed. Shoots and roots were oven‐dried at 60°C to record dry weights. Root mass ratio was calculated as the proportion of root dry weight to total plant dry weight.

Oven‐dried shoot and root samples were ground to a fine powder using a Geno/Grinder 2000 (Spex SamplePrep, Metuchen, NJ, USA). Shoot and root [P] were determined by digesting *c*. 100 mg of dry plant material in a hot concentrated nitric‐perchloric acid mixture (3 : 1), followed by quantification using the malachite green method (Motomizu *et al*., 1983). Leaf [N] was measured on *c*. 50 mg subsamples using a Vario Macro combustion analyser (Elementar Analysensysteme GmbH, Hanau, Germany). Shoot and root P contents were calculated as the product of shoot/root [P] and shoot/root dry weight. The leaf N : P ratio was determined as the ratio of [N] to [P] in leaves. The photosynthesis rate per unit of leaf P (PPUE) was calculated as the ratio of *A*_mass_ to leaf [P], while physiological PUE was expressed as the ratio of shoot dry weight to shoot [P] (Pang *et al*., 2018b).

### Statistical analyses

All parameters were analysed using a nested analysis of variance (ANOVA) in Genstat v.22.1 (VSN International Ltd, Hemel Hempstead, UK). The model accounted for accessions nested within species (species/accessions), with four replicates as the blocking structure. This approach enabled the evaluation of significant differences among *Cicer* species and accessions within species. When species effects were significant, the least significant difference (LSD) at *P* = 0.05 is reported in the figures. For significant differences among accessions within species, LSD_0.05_ values are presented in Supporting Information. Linear regression was used to model the correlation between intrinsic WUE and PNUE with *Cicer* species as a fixed factor using Genstat v.22.1. A regression model was chosen to best fit the data. Principal component analysis (PCA) was performed on 20 plant traits using the correlation matrix in OriginPro 2024b (OriginLab Corp., Northampton, MA, USA). PCA biplots display plant traits as PC factor loadings and species/accessions as PC scores. Additionally, Pearson's correlation analysis was conducted for the 20 plant traits, with the results visualized as a heat map generated in OriginPro 2024b.

## Results

### Plant height and branch number

**Fig. 1:**
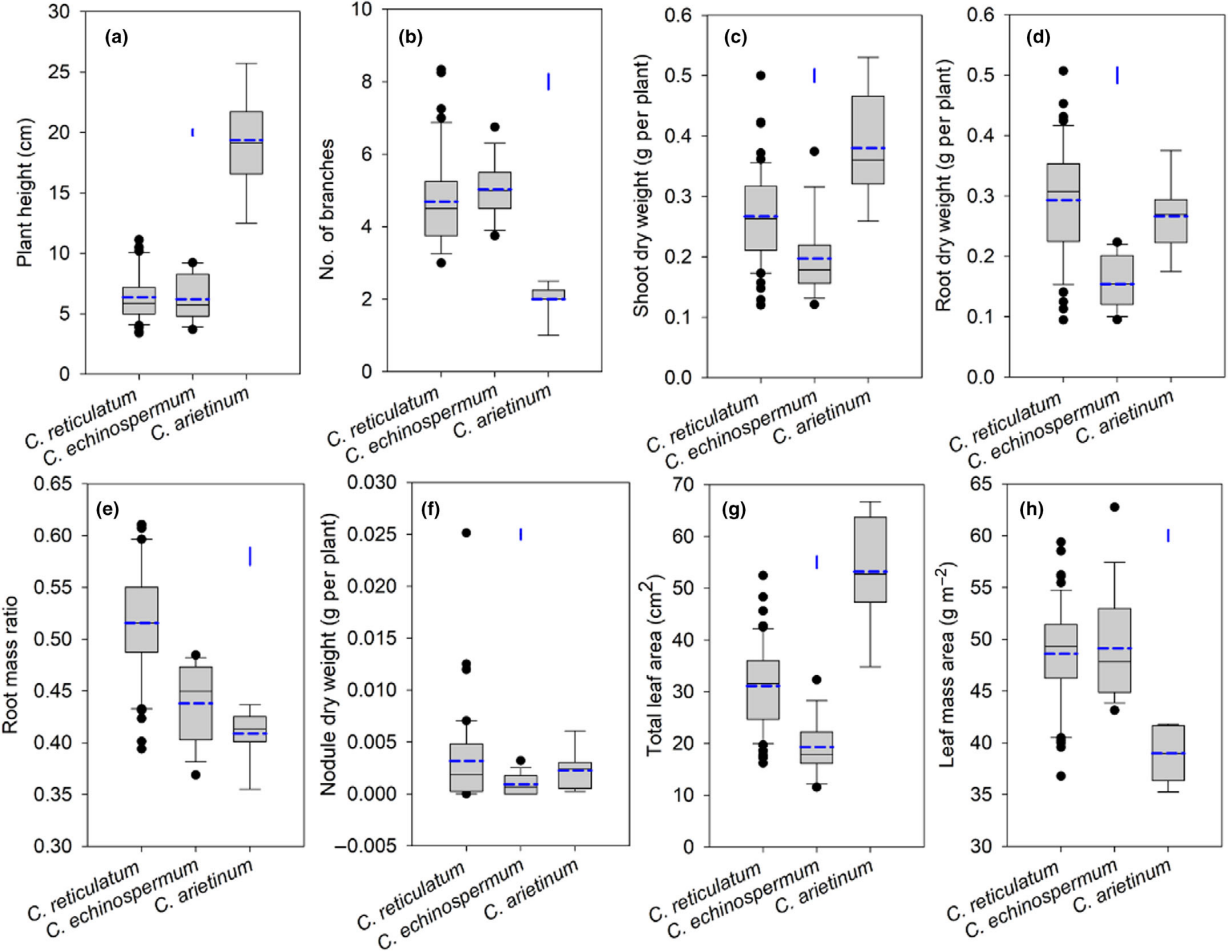
Box plots showing (a) plant height, (b) branch number, (c) shoot dry weight, (d) root dry weight, (e) root mass ratio, (f) nodule dry weight, (g) total leaf area, and (h) leaf mass area in three *Cicer* species – 54 *C. reticulatum*, 15 *C. echinospermum*, and seven *C. arietinum* accessions – grown under low‐P conditions for 9 wk in a temperature‐controlled glasshouse. Solid and dashed central bars in each box represent the median and mean, respectively. The box represents the interquartile range, the whiskers indicate the location of the most extreme data that are still within a factor of 1.5 of the upper or lower quartiles, and the black points are values that fall outside the whiskers. The blue vertical bars represent LSD_0.05_ between species.

We observed significant differences in plant height and branch number among *Cicer* species and accessions within species (all *P* < 0.001; Figs 1a,b, S1a,b). Both wild *Cicer* species were significantly shorter than cultivated chickpea (*P* < 0.001), with a mean plant height of 6.4 cm (range 3.4–11.1 cm) for *C. reticulatum* accessions, 6.2 cm (range 3.7–9.2 cm) for *C. echinospermum* accessions, and 19.4 cm (range 12.5–25.7 cm) for *C. arietinum* accessions. Interestingly, the wild species had significantly more branches, with a mean of 4.7 branches per plant for *C. reticulatum* and 5.0 branches per plant for *C. echinospermum*, compared with just 2.0 branches per plant for *C. arietinum*.

### Shoot and root dry weights and root mass ratio

Shoot and root dry weight varied significantly among species and accessions within species (all *P* < 0.001; Figs 1c,d, S1c,d). The mean shoot dry weight (DW) was greatest for *C. arietinum* (0.38 g per plant), followed by *C. reticulatum* (0.27 g per plant), and lowest in *C. echinospermum* (0.20 g per plant). Shoot DW varied more than four‐fold among 54 *C. reticulatum* accessions (range 0.12–0.50 g per plant), three‐fold among 15 *C. echinospermum* accessions (range 0.12–0.37 g per plant), and two‐fold among 7 *C. arietinum* accessions (range 0.26–0.53 g per plant). Root DW was greatest in *C. reticulatum* (0.29 g per plant), followed by *C. arietinum* (0.27 g per plant), and the lowest in *C. echinospermum* (0.15 g per plant). Root DW varied 5.7‐fold among *C. reticulatum* accessions (0.09–0.51 g per plant) and about two‐fold among *C. echinospermum* (0.10–0.22 g per plant) and *C. arietinum* (0.18–0.38 g per plant) accessions.

We found significant differences in root mass ratio among *Cicer* species and accessions within species (both *P* < 0.001; Figs 1e, S1e), with the highest mean value for *C. reticulatum* (0.52, range 0.40–0.61), followed by *C. echinospermum* (0.44, range 0.37–0.48) and *C. arietinum* (0.41, range 0.36–0.44).

### Nodule dry weight

Although the nodule dry weight per plant was, in general, low under low‐P conditions, there was a significant difference among *Cicer* species (*P* < 0.001; Fig. 1f), with *C. reticulatum* having the highest mean value (3.2 mg per plant), followed by *C. arietinum* (2.3 mg per plant), while *C. echinospermum* had the lowest mean value (0.9 mg per plant). The difference among accessions within each *Cicer* species was also significant (*P* < 0.001; ranging from 0 to 25 mg per plant; Fig. S1f).

### Total leaf area and leaf mass per area

We observed significant differences in total leaf area and LMA among *Cicer* species and accessions within species (all *P* < 0.001; Figs 1g,h, S1g,h). *Cicer arietinum* had the largest mean leaf area (53 cm^2^ per plant), followed by *C. reticulatum* (31 cm^2^ per plant), and the lowest was shown by *C. echinospermum* (19 cm^2^ per plant). Wild accessions exhibited a three‐fold difference in total leaf area (*C. reticulatum*, 16–53 cm^2^ per plant; *C. echinospermum*, 12–32 cm^2^ per plant), while *C. arietinum* accessions showed a two‐fold difference (35–67 cm^2^ per plant).

*Cicer reticulatum* and *C. echinospermum* had a significantly greater mean LMA (*c*. 49 g m^−2^ for both) than *C. arietinum* (39 g m^−2^), which varied 1.6‐fold among *C. reticulatum* accessions, 1.5‐fold among *C. echinospermum* accessions, and 1.2‐fold among *C. arietinum* accessions.

### Shoot and root P concentration and contents and leaf P concentration related to photosynthesis

**Fig. 2:**
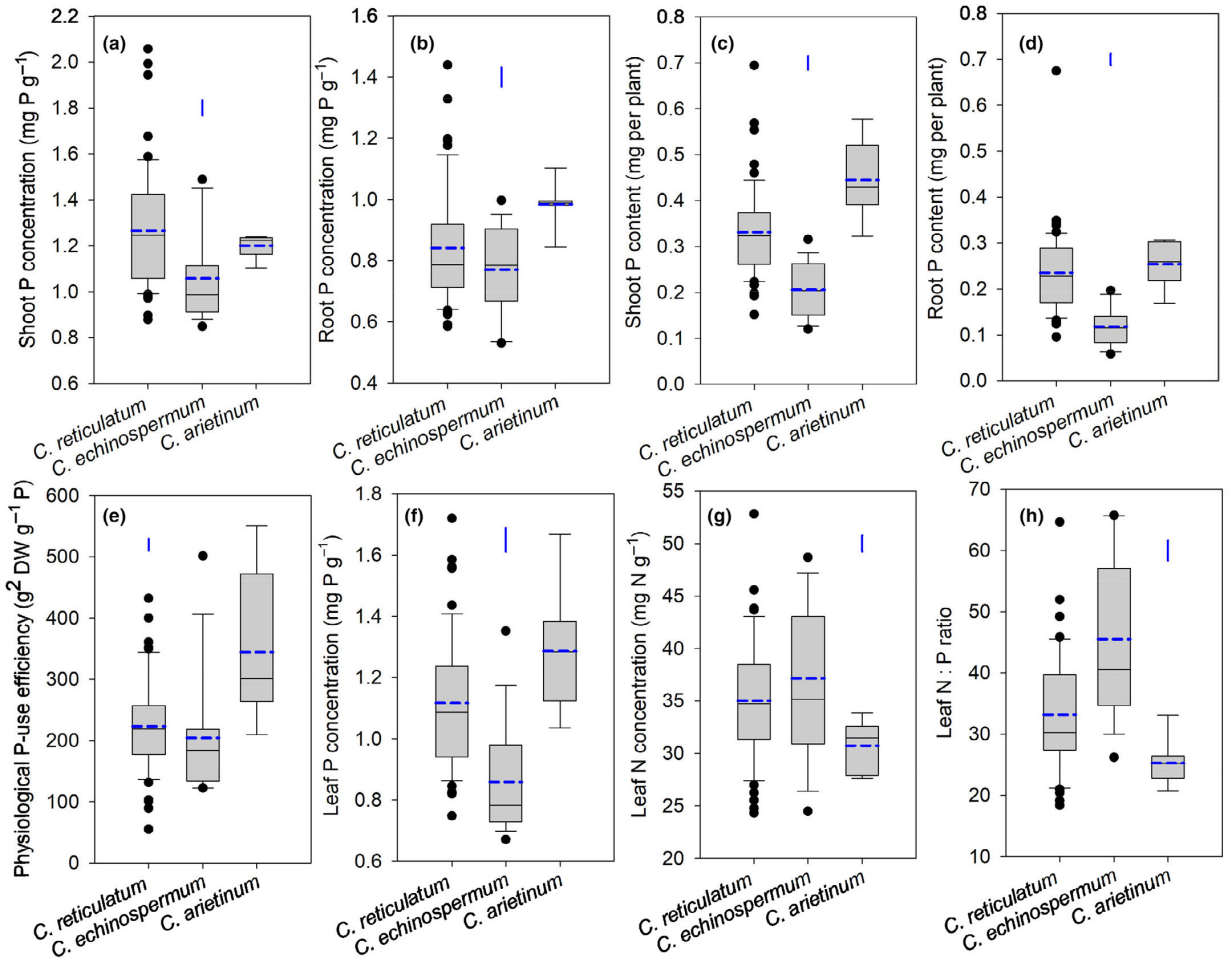
Box plots showing (a) shoot phosphorus (P) concentration, (b) root P concentration, (c) shoot P content, (d) root P content, (e) physiological P‐use efficiency, (f) P concentration ([P]) in the young fully expanded leaves for photosynthesis measurement, (g) leaf nitrogen (N) concentration and (h) leaf N : P ratio in three *Cicer* species – 54 *C. reticulatum*, 15 *C. echinospermum*, and seven *C. arietinum* accessions – grown under low‐P conditions for 9 wk in a temperature‐controlled glasshouse. Solid and dashed central bars in each box represent the median and mean, respectively. The box represents the interquartile range, the whiskers indicate the location of the most extreme data that are still within a factor of 1.5 of the upper or lower quartiles, and the black points are values that fall outside the whiskers. The blue vertical bars represent LSD_0.05_ between species.

Shoot and root [P], young fully expanded leaf [P], and shoot and root P contents significantly differed among *Cicer* species and accessions within species (all *P* < 0.001; Figs 2a−d,f, S2a−d,f). Mean shoot [P] was highest in *C. reticulatum* (1.27 mg P g^−1^), followed by *C. arietinum* (1.20 mg P g^−1^), and lowest in *C. echinospermum* (1.06 mg P g^−1^). The variation in shoot [P] was greater among the accessions within *C. reticulatum* (2.3‐fold, range 0.9–2.1 mg P g^−1^) and *C. echinospermum* (1.8‐fold, range 0.9–1.5 mg P g^−1^) than for *C. arietinum* (1.1‐fold, range 1.1–1.2 mg P g^−1^). Young fully expanded leaf [P] followed a similar trend to shoot [P].

Mean root [P] was highest in *C. arietinum* (0.98 mg P g^−1^), followed by *C. reticulatum* (0.84 mg P g^−1^), and lowest in *C. echinospermum* (0.77 mg P g^−1^). Variation in root [P] among accessions within species followed a similar trend to shoot [P], with *C. reticulatum* (2.4‐fold) and *C. echinospermum* (1.9‐fold) having greater variation than *C. arietinum* (1.3‐fold).

The mean shoot P content was greatest in *C. arietinum* (0.45 mg per plant), followed by *C. reticulatum* (0.33 mg per plant), and lowest in *C. echinospermum* (0.21 mg per plant). *Cicer arietinum* and *C. reticulatum* had a similar mean root P content, about two‐fold greater than that of *C. echinospermum*.

### Physiological P‐use efficiency

We observed significant variation in physiological PUE among *Cicer* species and accessions within species (both *P* < 0.001; Figs 2e, S2e). The mean physiological PUE was highest in *C. arietinum* (345 g^2^ DW g^−1^ P, range 210–551 g^2^ DW g^−1^ P), significantly lower in *C. reticulatum* (223 g^2^ DW g^−1^ P, range 55–432 g^2^ DW g^−1^ P) and lowest in *C. echinospermum* (204 g^2^ DW g^−1^ P, ranging from 122 to 502 g^2^ DW g^−1^ P).

### Leaf N concentration and N : P ratio

Leaf [N] and N : P ratio varied significantly among *Cicer* species and accessions within species (all *P* < 0.001; Figs 2g,h, S2g,h). Leaf [N] was highest in *C. echinospermum* (37.1 mg N g^−1^, range 24.5–48.7 mg N g^−1^), followed by *C. reticulatum* (35.0 mg N g^−1^, range 24.3–52.8 mg N g^−1^), and lowest in *C. arietinum* (30.7 mg N g^−1^, range 27.6–33.9 mg N g^−1^). The leaf N : P ratio followed the same trend.

### Leaf photosynthesis, stomatal conductance, intercellular CO_2_ concentration, and water‐use efficiency

**Fig. 3:**
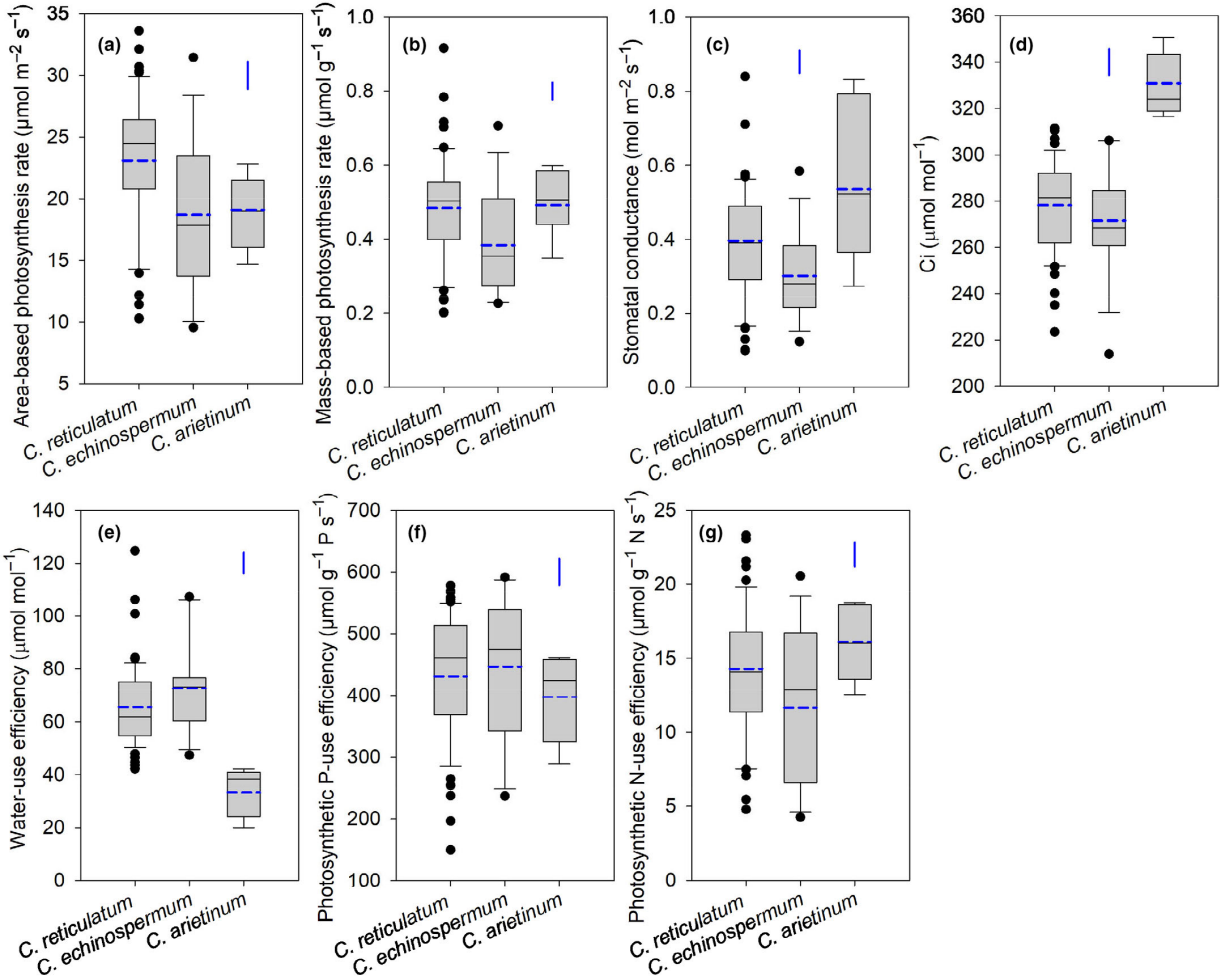
Box plots showing (a) area‐based and (b) mass‐based leaf photosynthesis rate, (c) stomatal conductance, (d) intercellular CO_2_ concentration (C_i_), (e) instantaneous water‐use efficiency, (f) photosynthetic P‐use efficiency, and (g) photosynthetic N‐use efficiency in three *Cicer* species – 54 *C. reticulatum*, 15 *C. echinospermum*, and seven *C. arietinum* accessions – grown under low‐P conditions for 9 wk in a temperature‐controlled glasshouse. Solid and dashed central bars in each box represent the median and mean, respectively. The box represents the interquartile range, the whiskers indicate the location of the most extreme data that are still within a factor of 1.5 of the upper or lower quartiles, and the black points are values that fall outside the whiskers. The blue vertical bars represent LSD_0.05_ between species.

Area‐ and mass‐based leaf photosynthesis rates, stomatal conductance, intercellular CO_2_ concentration, and intrinsic WUE differed significantly among *Cicer* species and accessions within species (all *P* < 0.001; Figs 3a−e, S3a−e). *Cicer reticulatum* had a significantly faster mean area‐based photosynthesis rate (23.1 μmol m^−2^ s^−1^) than *C. echinospermum* (18.8 μmol m^−2^ s^−1^) and *C. arietinum* (19.1 μmol m^−2^ s^−1^). The variation among accessions within *C. reticulatum* and *C. echinospermum* (both 3.3‐fold) was greater than that in *C. arietinum* (1.6‐fold). *Cicer reticulatum* and *C. arietinum* had similar mass‐based photosynthesis rates (both 0.49 μmol g^−1^ s^−1^), significantly greater than that of *C. echinospermum* (0.39 μmol g^−1^ s^−1^). Its variation among accessions within *C. reticulatum* (4.6‐fold) was greater than that of *C. echinospermum* (3.1‐fold) and *C. arietinum* (1.7‐fold).

The mean stomatal conductance was greatest in *C. arietinum* (0.54 mol m^−2^ s^−1^), followed by that of *C. reticulatum* (0.40 mol m^−2^ s^−1^), and it was lowest in *C. echinospermum* (0.30 mol m^−2^ s^−1^). Accession variation was greatest within *C. reticulatum* (8.4‐fold), followed by *C. echinospermum* (4.8‐fold) and *C. arietinum* (3.1‐fold). *Cicer arietinum* had a significantly higher mean C_i_ than *C. reticulatum* and *C. echinospermum*, with values ranging from 317 to 351 μmol mol^−1^ for *C. arietinum* accessions, from 223 to 311 μmol mol^−1^ for *C. reticulatum* accessions, and from 214 to 306 μmol mol^−1^ for *C. echinospermum* accessions.

*Cicer reticulatum* and *C. echinospermum* had similar mean intrinsic WUE values (65 and 72 μmol mol^−1^, respectively), significantly greater than those of *C. arietinum* (33 μmol mol^−1^). All *C. reticulatum* and *C. echinospermum* accessions had greater intrinsic WUE (42–125 and 47–107 μmol mol^−1^, respectively) than *C. arietinum* accessions (20–42 μmol mol^−1^).

### Leaf photosynthetic P‐use and N‐use efficiencies

Leaf PPUE and PNUE varied significantly among *Cicer* species and accessions within species (*P* < 0.001; Figs 3f,g, S3f,g). *Cicer echinospermum* had the highest mean PPUE (452 μmol g^−1^ P s^−1^), followed by *C. reticulatum* (432 μmol g^−1^ P s^−1^), and it was lowest in *C. arietinum* (397 μmol g^−1^ P s^−1^). Accession variation for PPUE was greatest within *C. reticulatum* (3.8‐fold), followed by *C. echinospermum* (2.5‐fold) and *C. arietinum* (1.6‐fold). Interestingly, *c*. 50% of the studied wild accessions (26 of *C. reticulatum* and eight of *C. echinospermum*) had PPUE values exceeding the highest value observed in *C. arietinum* (i.e. ICC4814).

Leaf PNUE followed an opposite trend to that of PPUE, with the highest mean value for *C. arietinum* (16.1 μmol g^−1^ N s^−1^), followed by *C. reticulatum* (14.2 μmol g^−1^ N s^−1^) and *C. echinospermum* (12.0 μmol g^−1^ N s^−1^). Accession variation among *C. reticulatum* (4.9‐fold) and *C. echinospermum* (4.4‐fold) was significantly greater than that among *C. arietinum* (1.5‐fold).

### Correlations among traits

**Fig. 4:**
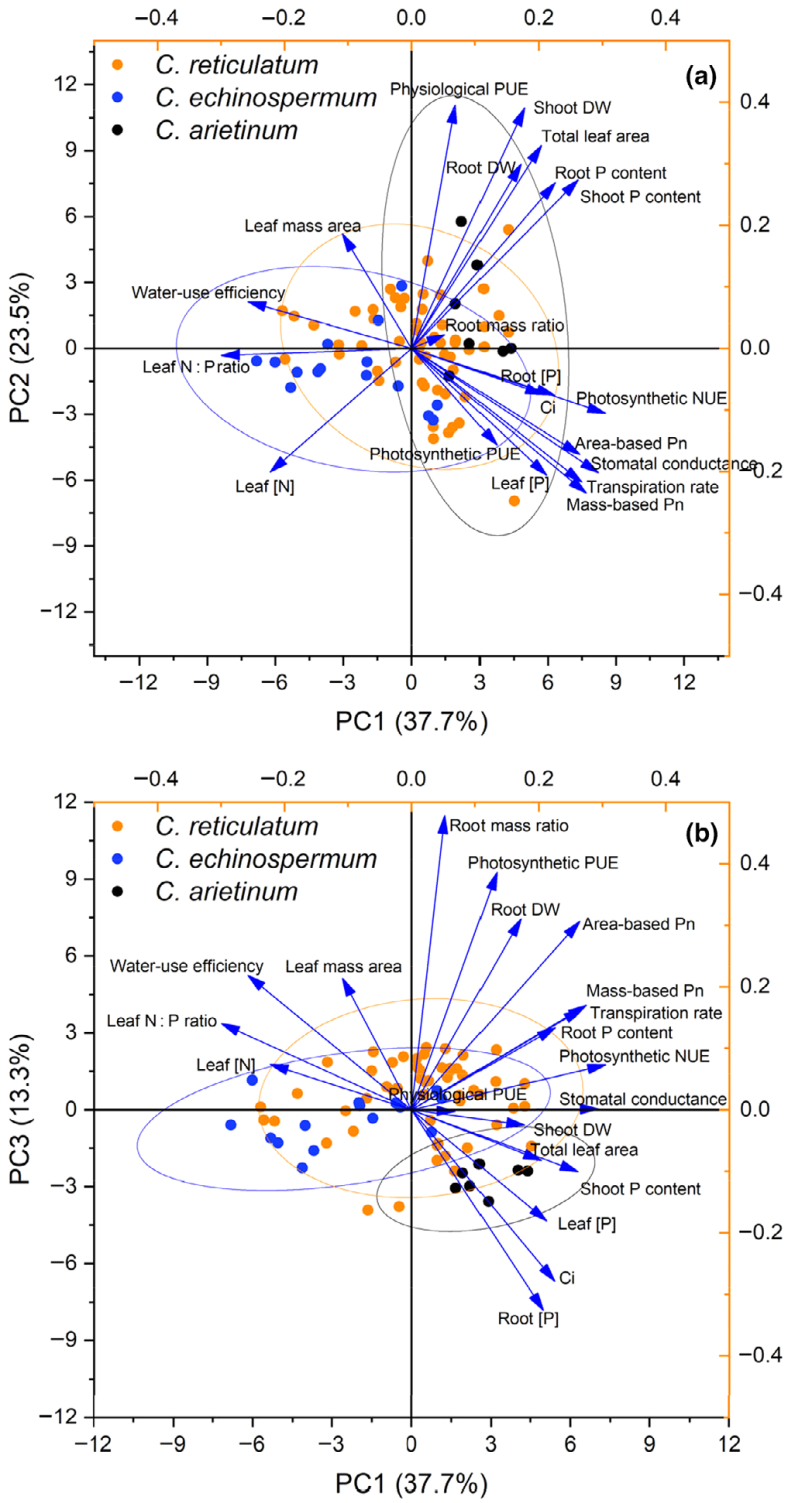
Principal component analysis of 20 plant parameters for 54 *Cicer reticulatum*, 15 *C. echinospermum*, and seven *C. arietinum* accessions grown under low‐P conditions for 9 wk in a temperature‐controlled glasshouse. Biplot vectors are trait factor loadings, and the position of each accession of *C. reticulatum* (orange), *C. echinospermum* (blue), and *C. arietinum* (black) is shown for principal components: (a) PC1 (38% of the variability) vs PC2 (24% of the variability), and (b) PC1 vs PC3 (13% of the variability). C_i_, intercellular CO_2_; Pn, photosynthesis rate; PUE, phosphorus‐use efficiency; NUE, nitrogen‐use efficiency.

The PCA analysis based on 20 plant traits for 54 *C. reticulatum*, 15 *C. echinospermum*, and 7 *C. arietinum* accessions revealed that three principal components explained 75% of the total variance (Fig. 4a,b; Table 2). The first component (PC1), which accounted for 37.7% of the variation, comprised primarily area‐based and mass‐based photosynthesis rates, leaf stomatal conductance, transpiration rate, leaf N : P ratio, shoot P content, PNUE, and instantaneous intrinsic WUE, whereas PC2, accounting for 23.5% of the variation, reflected variation in shoot and root dry weight, total leaf area, and physiological PUE, while PC3 (13.3%) comprised root dry weight, root mass ratio, area‐based leaf photosynthesis rate, C_i_, root [P] and PPUE.

**Table 2:** Variable loading scores of 20 plant parameters for 54 *Cicer reticulatum*, 15 *C. echinospermum*, and seven *C. arietinum* accessions, and the proportion of variation for each principal component.

	PC1	PC2	PC3
Shoot dry weight	0.18	**0.39**	−0.02
Root dry weight	0.17	**0.30**	**0.31**
Root mass ratio	0.05	0.02	**0.48**
Total leaf area	0.20	**0.33**	−0.08
Leaf mass per area	−0.11	0.19	0.21
Area‐based Pn	**0.26**	−0.17	**0.31**
Mass‐based Pn	**0.27**	−0.24	0.17
Stomatal conductance	**0.29**	−0.20	0.00
Transpiration rate	**0.27**	−0.22	0.16
C_i_ (intercellular CO_2_ concentration)	0.23	−0.08	−**0.28**
Leaf [N]	−0.22	−0.20	0.07
Leaf [P]	0.21	−0.21	−0.18
Root [P]	0.21	−0.08	−**0.33**
Leaf N : P	−**0.30**	−0.01	0.14
Shoot P content	**0.26**	0.27	−0.10
Root P content	0.23	0.27	0.13
Photosynthetic NUE	**0.30**	−0.11	0.07
Photosynthetic PUE	0.13	−0.16	**0.39**
Water‐use efficiency	−**0.26**	0.08	0.22
Physiological PUE	0.07	**0.40**	0.00
Variability (%)	38	24	13
Cumulative variability (%)	38	62	75

The largest variable loading scores in the three components are in bold. NUE, nitrogen‐use efficiency; Pn, photosynthesis rate; PUE, phosphorus‐use efficiency.

**Fig. 5:**
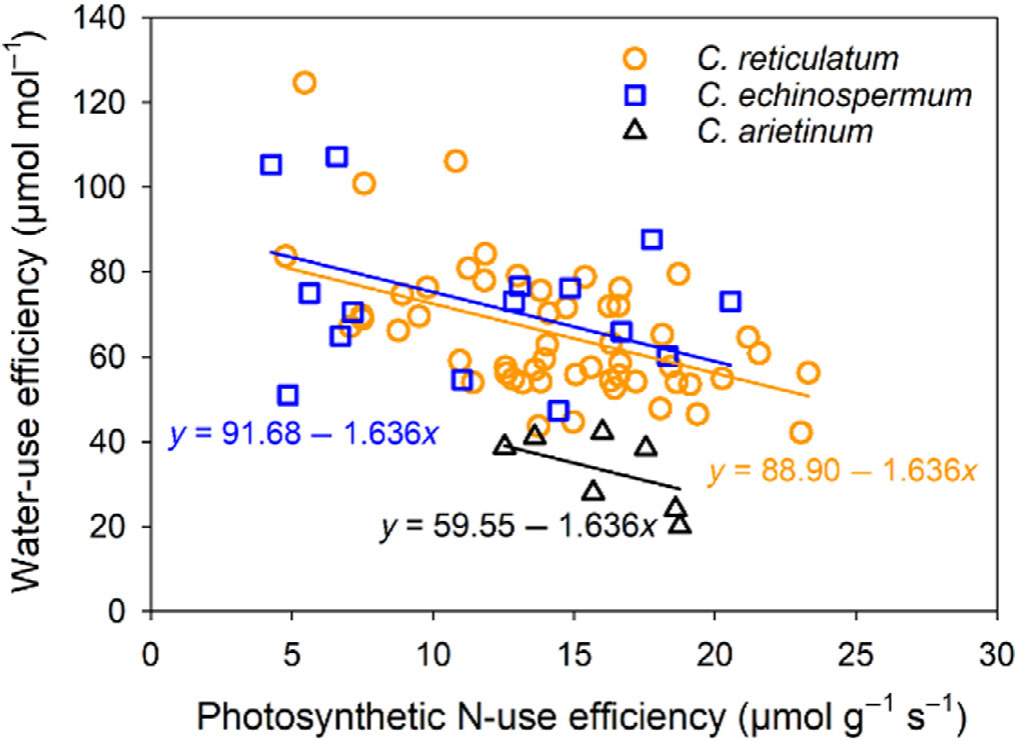
Linear regression model (solid lines) illustrating the relationship between intrinsic water‐use efficiency against photosynthetic nitrogen‐use efficiency at the leaf level in 76 *Cicer* accessions belonging to three *Cicer* species, including 54 *C. reticulatum* (orange circle), 15 *C. echinospermum* (blue square), and seven *C. arietinum* (dark triangle) accessions. The model explained 44% of the variance. All plants were grown under low‐phosphorus conditions for 9 wk in a temperature‐controlled glasshouse.

**Fig. 6:**
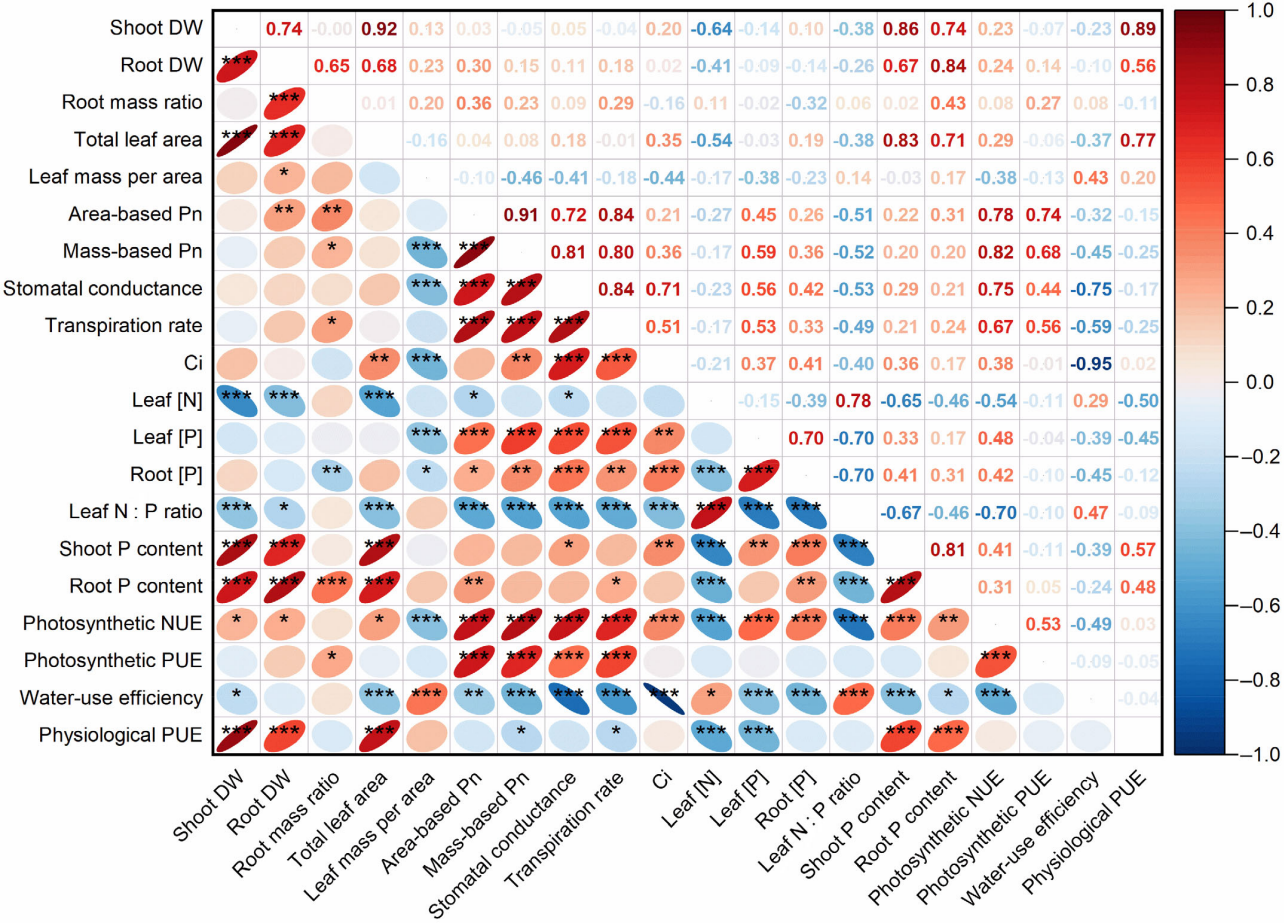
Heat map showing Pearson's correlation matrix for 20 plant traits in 76 *Cicer* accessions (54 *C. reticulatum*, 15 *C. echinospermum*, and 7 *C. arietinum*). The lower triangle shows ellipses with significant marks, while the upper triangle shows the correlation coefficients. *, *P* < 0.05; **, *P* < 0.01; ***, *P* < 0.001. Plant trait abbreviations: DW, dry weight; C_i_, intercellular CO_2_; Pn, photosynthesis rate; [N], nitrogen concentration; [P], phosphorus concentration; NUE, nitrogen‐use efficiency; PUE, phosphorus‐use efficiency.

Biplots in Fig. 4 and Pearson's correlation analysis (Figs 5, 6) revealed strong correlations among some of the 20 plant traits. Across all 79 accessions, we found a significant negative correlation between intrinsic WUE and leaf PNUE (*r* = −0.49, *P* < 0.001; Figs 4a, 6). Linear regression of intrinsic WUE against PNUE indicated separate intercepts for each *Cicer* species (*P* < 0.001) and a common slope, accounting for 44% of the variance (Fig. 5). Thus, domesticated chickpea had a much lower intrinsic WUE than wild *Cicer* species, with a similar trade‐off against PNUE as indicated by the common slope.

Across all species, LMA was negatively correlated with stomatal conductance (*r* = −0.41, *P* < 0.001), C_i_ (*r* = −0.44, *P* < 0.01), leaf [P] (*r* = −0.38, *P* < 0.001), and PNUE (*r* = −0.38, *P* < 0.001), but positively correlated with intrinsic WUE (*r* = 0.43, *P* < 0.001; Fig. 6). Leaf [P] was positively correlated with leaf transpiration rate (*r* = 0.56, *P* < 0.001) across species. Leaf intrinsic WUE was negatively correlated with stomatal conductance (*r* = −0.75, *P* < 0.001) and C_i_ (*r* = −0.95, *P* < 0.001) across species.

## Discussion

This study is the first comprehensive comparison of growth, leaf gas exchange, PPUE, and PNUE across three *Cicer* species under low‐P conditions. The present findings fully support our first hypothesis, revealing significant variation in leaf gas exchange characteristics, PPUE, and PNUE among *Cicer* species and accessions within species. Pang *et al*. (2018b) reported genotypic variation in plant growth, shoot P content, and physiological PUE in a unique chickpea reference set of 266 *C. arietinum* accessions with diverse genetic backgrounds from 29 countries and variation in photosynthesis rate and PPUE among a subset of 100 accessions at the seedling stage under low‐P conditions. Kumar *et al*. (2024) identified significant genotypic differences in plant height, total dry weight, seed yield, seed size, and harvest index at physiological maturity among 288 chickpea accessions grown under low‐ and high‐P conditions in a 2‐yr field study. Building on these findings, our study highlights the greater genetic diversity in *C. reticulatum* and *C. echinospermum* than in domesticated chickpea, offering new insights into plant growth, photosynthetic traits, PPUE, and PNUE in wild *Cicer* species under low‐P conditions. The significant genetic diversity of wild *Cicer* species can be harnessed to develop chickpea varieties with enhanced P‐use efficiency.

The present study demonstrates that wild *Cicer* species exhibited greater variation in leaf gas exchange characteristics and PPUE than cultivated chickpea under low‐P conditions, fully supporting our second hypothesis. Notably, this study revealed extensive variation among accessions of wild *Cicer* species relative to domesticated chickpea under low‐P conditions. For example, we observed a 3.3‐fold variation in area‐based leaf photosynthesis rate within 54 *C. reticulatum* accessions and 15 *C. echinospermum* accessions. *Cicer reticulatum* accessions exhibited a 4.6‐fold variation in mass‐based leaf photosynthesis rate and a 3.8‐fold variation in PPUE. Stomatal conductance varied 8.4‐fold within *C. reticulatum* and 4.8‐fold within *C. echinospermum*. This variation in wild *Cicer* species was notably greater than that observed among *C. arietinum* accessions in the present study, which only showed 1.6‐fold, 1.7‐fold, 3.1‐fold, and 1.6‐fold variation in area‐based photosynthesis, mass‐based photosynthesis, stomatal conductance, and PPUE, respectively. This genotypic variation in wild *Cicer* species exceeded that observed in 100 *C. arietinum* accessions from a genetically diverse chickpea reference set, grown under low‐P conditions similar to those of the present study, which only showed a 2.3‐fold variation in area‐based photosynthesis, a 2.8‐fold variation in mass‐based photosynthesis, a 2.9‐fold variation in stomatal conductance, and a 2.5‐fold variation in PPUE (Pang *et al*., 2018b). The more pronounced variation observed in wild *Cicer* species in this study was associated with a significantly greater number of polymorphic loci in *C. reticulatum* and *C. echinospermum* than in *C. arietinum* (von Wettberg *et al*., 2018). These findings underscore the potential of wild *Cicer* species as valuable genetic resources providing donor genes to enhance leaf gas exchange characteristics and PPUE in chickpea breeding programmes. However, it is important to recognise that a higher photosynthetic capacity at the leaf level does not necessarily translate into a faster growth rate (Condon *et al*., 2004). Domestication typically leads to increased plant growth and biomass/yield production, such as in cotton (Lei *et al*., 2024) and chickpea (Berger *et al*., 2020; Marques *et al*., 2020). In the present study, although *C. reticulatum* exhibited faster leaf photosynthetic rates, this advantage was offset by its overall slower growth rates and lower biomass accumulation. Our goal was to identify germplasms with enhanced leaf photosynthetic rate and PPUE with the ultimate aim to integrate these traits into modern cultivars to further increase the whole canopy photosynthate accumulation and increase yield potential while reducing P fertiliser input. By incorporating these traits into domesticated chickpea, new varieties with greater PPUE under low‐P conditions will be developed, therefore enhancing resilience to P‐deficient soils.

Our third hypothesis, that wild *Cicer* species exhibit greater PPUE than cultivated *Cicer*, was fully supported. This study revealed that 26 *C. reticulatum* accessions and 8 *C. echinospermum* accessions displayed greater PPUE values exceeding the highest value observed in *C. arietinum*. These findings significantly expand the available germplasm resource for enhancing PPUE in chickpea breeding programmes. The higher PPUE in *C. reticulatum* than in *C. arietinum* can be attributed to its 13% lower leaf [P] while maintaining a similar mass‐based photosynthesis rate. Although *C. echinospermum* exhibited the lowest photosynthesis rates and leaf [P] among the three *Cicer* species, the lower leaf [P] (34%) was more pronounced than the lower leaf photosynthesis rate (20%) relative to *C. arietinum*, resulting in a higher PPUE. These findings contrast with the effects of domestication on PPUE in soybean, where domesticated soybean exhibits faster photosynthesis rates and lower leaf [P] than its ancestors (Chen *et al*., 2024).

Leaf PPUE is defined as the photosynthesis rate per unit leaf P. Leaf photosynthesis rate is influenced by factors such as [CO_2_] at the carboxylation site and photosynthetic capacity (e.g. ribulose 1,5‐bisphosphate (RuBP) carboxylation rate and electron transport rate for RuBP regeneration). Differences in these factors and leaf [P] likely explain the observed variations in PPUE in response to domestication. For instance, we found that *C. reticulatum* and *C. echinospermum* accessions had a significantly lower C_i_ than *C. arietinum*. This contrasts with findings in soybean, where domesticated soybean exhibits lower C_i_ and chloroplast CO_2_ (C_c_) but higher mesophyll conductance and faster photosynthesis rates than its wild ancestor (*Glycine soja*) (Pelech *et al*., 2025). A low C_i_ value reflects either relatively low stomatal conductance, relatively high photosynthetic capacity (i.e. the biochemical capacity for photosynthesis per unit leaf area, commonly represented by maximum carboxylation and electron transport rates), or a combination of both (Condon *et al*., 2004). The significant positive correlation between C_i_ and stomatal conductance in the present study suggests that the lower C_i_ in wild *Cicer* species than in *C. arietinum* was at least partly due to their significantly lower stomatal conductance.

Our results also revealed a negative correlation between C_i_ and LMA across the three *Cicer* species. The higher LMA observed in both wild *Cicer* species likely reflects thicker leaves and/or denser cellular structures than in *C. arietinum*. Thicker leaves are associated with longer diffusion pathways for CO_2_ from the leaf surface to mesophyll cells, typical of scleromorphic leaves (Lambers *et al*., 2010; Onoda *et al*., 2017). Consequently, the thicker leaves of *C. reticulatum* and *C. echinospermum* likely contribute to their lower C_i_ values, ultimately influencing photosynthesis rate.

The lower C_i_ values in wild *Cicer* species may indicate a relatively greater photosynthetic capacity, supported by our findings that leaf [N] in both wild species was significantly greater than that in cultivated chickpea. This pattern aligns with the findings in the tree *Ficus insipida*, which exhibits a significant negative correlation between C_i_ and leaf [N] (Cernusak *et al*., 2007). Nitrogen plays a crucial role in photosynthesis, comprising major components: soluble protein dominated by Rubisco, and proteins in the thylakoid membranes of the chloroplast (Evans, 1989). Strong correlations between Rubisco [N] and total leaf [N] have been observed in various crop species, including *Triticum aestivum*, *Oryza sativa*, *Spinacia oleracea*, *Phaseolus vulgaris*, and *Alocasia macrorrhiza*, although the specific activity of Rubisco and N partitioning into it vary among species (Evans, 1989). The higher leaf [N] in wild *Cicer* species suggests a potential for increased quantity and/or activity of Rubisco, contributing to enhanced photosynthetic capacity. This aligns with findings in dry‐site tree species, where a higher leaf [N] allows for a faster area‐based photosynthesis rate at lower stomatal conductance, reducing water loss compared with that in wet‐site tree species (Wright *et al*., 2001). The significantly lower stomatal conductance in wild *Cicer* species likely restricts water loss, enhancing their WUE. However, it remains unclear whether the three *Cicer* species differ in N partitioning to Rubisco or in the specific activity of this enzyme. Further research is needed to elucidate these aspects, such as measuring leaf Chl concentration and generating the response curves of photosynthesis to C_i_ (A−C_i_ curves) from which the maximum carboxylation rate and maximum electron transport rate can be derived. Those parameters would allow us to estimate N allocation to three major components associated with photosynthesis, that is, light harvesting, bioenergetics, and Rubisco.

While both wild *Cicer* species exhibited higher PPUE and lower leaf [P] than domesticated chickpea, there was no significant correlation between leaf PPUE and [P] across the three *Cicer* species, consistent with the findings in domesticated chickpea (Wen *et al*., 2022). To date, research on the detailed mechanisms underlying high PPUE, particularly in crops, remains limited. We propose two possible biochemical explanations for the higher PPUE in wild *Cicer* species than in domesticated *Cicer* in low‐P environments. First, the difference may stem from variation in P allocation within leaf cells. Many Proteaceae species from severely P‐impoverished soils in south‐western Australia and South Africa preferentially allocate foliar P to mesophyll cells rather than epidermal cells, enhancing their PPUE as an adaptive mechanism to low‐P habitats (Hawkins *et al*., 2008; Hayes *et al*., 2018). However, our previous study in chickpea shows no differences in cellular [P] between mesophyll and epidermis cells among four cultivated chickpea accessions with contrasting PPUE (Wen *et al*., 2022). Second, the higher PPUE in wild *Cicer* accessions may result from optimized allocation to foliar P fractions. Unlike in *Cicer*, domestication increases PPUE in soybean by enhancing P allocation to P‐containing metabolites (Chen *et al*., 2024). Similarly, Wang *et al*. (2024) reported increased PPUE across 10 domesticated crops due to greater P allocation to metabolite P and reduced allocation to lipid P and inorganic P. However, that study only evaluated one wild–cultivated pair for each species, limiting its generalizability. In *C. arietinum*, our earlier work shows that a higher PPUE is associated with reduced allocation to organic and metabolite P and increased allocation to nucleic acid P (Wen *et al*., 2022). Therefore, differences in P allocation within leaf cells and foliar P fractionation between wild and domesticated *Cicer* species warrant further investigation.

Both wild *Cicer* species exhibited lower PNUE than domesticated chickpea did, consistent with the results of Marques *et al*. (2020), who found significantly lower PNUE in *C. reticulatum* under N‐sufficient conditions. In the present study, we attribute the lower PNUE in *C. reticulatum* to a similar mass‐based photosynthesis rate but higher [N] than that in *C. arietinum*, and a lower mass‐based photosynthesis rate and higher [N] in *C. echinospermum*. This trend was partly explained by the significantly higher LMA in wild *Cicer* species, as evidenced by a negative correlation between PNUE and LMA among the three species. This finding aligns with the results of an N fertilization study by Marques *et al*. (2020), who reported a similar correlation between PNUE and LMA in *C. reticulatum* and domesticated chickpea. Additional support comes from broader datasets of noncrop species, such as those analysed by Onoda *et al*. (2017), showing that species with higher LMA tend to have lower PNUE, attributed to the allocation of a smaller fraction of leaf N to photosynthetic proteins and a greater fraction to cell walls. Similarly, cotton domestication enhances PNUE, with domesticated accessions allocating more N to the photosynthetic machinery, including light harvesting, bioenergetics, and Rubisco, while reducing absolute and relative N allocations to nonphotosynthetic N pools, compared with their wild counterparts (Lei *et al*., 2021).

Leaf [N] and [P] in *Cicer* species in the present study were evidently affected by different factors. For leaf [P], a significant negative correlation between leaf [P] and LMA suggests that higher LMA of the wild *Cicer* species contributed to their lower [P]. This finding is consistent with research showing a positive correlation between leaf [P] and specific leaf area (the reciprocal of LMA) across 79 perennial species from diverse environments in eastern Australia (Wright *et al*., 2001). Thicker leaves typically contain a greater proportion of structural tissues or compounds, such as sclerenchyma or cuticles; sclerenchyma comprises cells with thick walls that are low in P‐rich components and high in lignin and cellulose (Lambers *et al*., 2010). Furthermore, a positive correlation between leaf transpiration rate and leaf [P] suggests a role of leaf transpiration and stomatal conductance in P uptake across the three *Cicer* species. This finding supports and expands previous findings in domesticated chickpea grown in low‐P sandy soils (Pang *et al*., 2018b). The lower stomatal conductance in both wild *Cicer* species likely contributed to their lower leaf [P], with *C. echinospermum* having the lowest stomatal conductance and, consequently, the lowest leaf [P] among the three species. Additionally, leaf [P] differences may reflect the distinct native habitats of these species. The soils where *C. reticulatum* occurs tend to be more fertile and alkaline than those where *C. echinospermum* is found (von Wettberg *et al*., 2018). Nodule formation requires a significant amount of P investment to support their nitrogenase activity (Raven, 2012); however, the nodulation under low‐P conditions was generally low in the present study. *Cicer echinospermum*, with the lowest nodule dry weight per plant, had the lowest shoot [P] and leaf [P]. Therefore, the lower leaf [P] in both *C. reticulatum* and *C. echinospermum* than in domesticated *Cicer* would not primarily be due to nodule P consumption in the present study.

The lack of a positive correlation between leaf [N] and transpiration rate across the three *Cicer* species in the present study suggests that mass flow played a minor role in N acquisition. This contrasts with many studies highlighting the importance of mass flow in N uptake (Tinker & Nye, 2000; Matimati *et al*., 2013). However, it is possible that while mass flow transports nitrate to the soil–root interface, plants tightly regulate N uptake by controlling transport activity at the root level. For example, *Hakea prostrata* maintains a stable leaf [N] (20 mg N g^−1^ dry weight), regardless of nitrate supply level to the roots, indicating a tightly regulated N acquisition system (Prodhan *et al*., 2016). This regulation is considered an adaptive strategy for survival in low‐P environments, as reduced nitrate acquisition and assimilation constrain protein synthesis, therefore reducing P demand for rRNA (Prodhan *et al*., 2016). In Proteaceae, leaf soluble protein concentration is only 10–20% of that in *Arabidopsis thaliana*, yet their photosynthesis rates are similar (Sulpice *et al*., 2014). Although the activities of Rubisco and other enzymes are significantly lower on a fresh weight basis than those in *A. thaliana*, mature leaves of the Proteaceae species exhibit a 2.5‐fold higher Rubisco activity when expressed on a soluble protein basis, suggesting a preferential allocation of N to key photosynthetic enzymes, optimising function despite a reduced overall protein level (Sulpice *et al*., 2014). It is worth noting that while leaf [N] in all accessions of three *Cicer* species exceeded the critical threshold (23 mg N g^−1^ dry weight) for optimum chickpea growth, leaf [P] was below the critical level (2.4 mg P g^−1^ dry weight) (GRDC, 2017). Therefore, the lower leaf [N] in *C. arietinum* than that in wild *Cicer* species may reflect a tighter control of N acquisition, potentially as a strategy to reduce its P demand under low‐P conditions. Further investigation is needed to explore differences in N fractionation within leaves of the three *Cicer* species.

Since leaf [N] and [P] were affected by different factors, our study shows that the higher leaf [N] in both wild *Cicer* species was accompanied by significantly lower leaf [P] than that in domesticated chickpea, leading to a significantly higher leaf N : P ratio. Hidaka & Kitayama (2009) found a positive linear correlation between PPUE and N : P ratio across a broad set of tree and shrub species. Similarly, we observed that both wild *Cicer* species, with a higher leaf N : P ratio, also exhibited greater PPUE than domesticated chickpea; however, the correlation between PPUE and leaf N : P was not significant across the three *Cicer* species. Compared with wild *Cicer* species, *C. arietinum* had a greater leaf [P] relative to [N]. According to the growth rate hypothesis, species with rapid growth rates tend to have a low N : P ratio, as fast growth requires a proportionally greater P investment than N, particularly in P‐rich ribosomal RNA (rRNA), to meet the protein synthesis demand required for rapid growth (Reich *et al*., 2010; Rees & Raven, 2021; Lambers, 2022). However, in the present study, the leaf N : P ratio was measured in young but mature nongrowing leaves. Since those nucleic acids, particularly rRNA, constitute a major organic P pool, the higher leaf [P] in *C. arietinum* might indicate a faster protein turnover. By contrast, both wild *Cicer* species had lower leaf [P] but higher leaf [N], suggesting that they may prioritise maintaining high concentrations of key N‐rich enzymes rather than rapidly synthesising new proteins, therefore requiring less rRNA (and thus less P) to support their slower protein turnover. Therefore, the variation in leaf [P] and, consequently, rRNA levels among the three *Cicer* species likely reflects differences in protein turnover rather than a direct association with plant growth rates. The faster protein turnover rate in *C. arietinum* may enhance its proteome flexibility, presumably allowing it to better acclimate to environmental challenges such as low P availability (Lambers, 2022). Further research is warranted to explore this correlation in greater depth.

Our fourth hypothesis, that wild *Cicer* species exhibit a more conservative WUE as a trade‐off for PNUE, was fully supported. In this study, all 54 *C. reticulatum* accessions and 15 *C. echinospermum* accessions had significantly higher intrinsic WUE than the 7 *C. arietinum* accessions. Moreover, WUE was positively correlated with LMA and negatively correlated with stomatal conductance and C_i_. These findings align with those of Marques *et al*. (2020), who reported that *C. reticulatum* comprises phenotypes that have lower water loss, higher LMA, and lower stomatal conductance than domesticated chickpea. Z. Lei *et al*. (2023) showed that cotton domestication reduces WUE by altering stomatal traits, particularly by increasing the size of abaxial stomata. The higher LMA and lower stomatal conductance in wild *Cicer* species likely reflect adaptations to frequent drought stress in their native habitats in eastern Türkiye. However, while intrinsic leaf‐level WUE was higher in wild *Cicer*, the whole‐plant WUE might differ due to the greater total leaf area in domesticated chickpea, which warrants further investigation, particularly under low‐P conditions.

Our study demonstrates a significant negative correlation between WUE and PNUE across all three *Cicer* species, suggesting that the higher WUE in both wild *Cicer* species came at the expense of PNUE compared with that in domesticated chickpea. This trade‐off between PNUE and WUE has been observed in noncrop plants, such as in five species of California evergreens (Field *et al*., 1983), a broad range of noncrop species (Wright *et al*., 2001), and four tree species, including *Banksia grandis*, *Eucalyptus patens*, *Corymbia calophylla*, and *E. marginata* (Zhou *et al*., 2024). The least‐cost economic theory of photosynthesis suggests that water and N are mutually substitutable resources for achieving a given carbon assimilation gain, and higher leaf [N] is typically associated with higher carboxylation capacity and tighter stomatal regulation of transpiration, leading to higher leaf‐level WUE and a more conservative water‐use strategy in dryland natural ecosystems (Wright *et al*., 2003; Prentice *et al*., 2014; Paillassa *et al*., 2020; Querejeta *et al*., 2022). In our study, the higher WUE in both wild *Cicer* species was primarily attributed to their significantly lower stomatal conductance than that in *C. arietinum*. These results support and extend earlier findings in crops by van den Boogaard *et al*. (1995), who observed that high WUE is associated with low PNUE in wheat (*Triticum aestivum*) under high irradiance, where reduced stomatal conductance enhances WUE; however, no such trade‐off is observed when differences in WUE arise from a faster photosynthesis rate driven by higher [N], such as under low irradiance and high water and N supply.

### Concluding remarks

Our study demonstrates significant variation in shoot and root dry weight (DW), root mass ratio, shoot and root P contents, physiological PUE, leaf gas exchange characteristics, PPUE, and PNUE among three *Cicer* species (*C. reticulatum*, *C. echinospermum*, and *C. arietinum*) and accessions within each species under low‐P conditions. Given such genotypic variation among wild species/accessions, caution should be exercised when drawing conclusions from early studies comparing domesticated cultivars and their wild relatives, particularly when only 1–2 accessions of wild relatives are involved. Both wild *Cicer* species demonstrated greater PPUE than the cultivated *C. arietinum*. They exhibited significantly lower stomatal conductance and higher LMA, resulting in lower C_i_ and higher WUE than those of *C. arietinum*. We surmise that higher leaf [N] in both wild *Cicer* species may lead to increased levels of Rubisco, enhancing their photosynthetic capacity and compensating for their lower stomatal conductance than that in *C. arietinum*. However, the increased WUE in both wild *Cicer* species was traded off against a lower PNUE relative to that of domesticated chickpea. Enhancing leaf PPUE may reduce dependence on P fertilizers. Further exploration of the mechanisms underlying higher PPUE in wild *Cicer* is needed. Our findings underscore the significant potential of wild *Cicer* species as valuable genetic resources for providing donor genes to enhance PPUE in chickpea improvement programmes. Extrapolating these results beyond the vegetative stage requires further experimentation. To make the selection of accessions more robust, the identified accessions should be verified for their desirable traits under different soil types and environmental conditions across multiple field sites in the future.

## Competing Interests

None declared.

## Author Contributions

JP, HL, JB, UM and KHMS designed the study; JP, SL, KDS and WZ performed the experiment and collected the data; JP and JB analyzed the data; JP and HL discussed the data; JP led the writing of the manuscript. All authors, including RKV, contributed critically to the drafts and approved the final version for publication.

## Disclaimer

The New Phytologist Foundation remains neutral with regard to jurisdictional claims in maps and in any institutional affiliations.

## Supporting information


**Dataset S1** Raw data for Figs 1–6 and S1–S3.

**Fig. S1** Plant height, branch number, shoot dry weight, root dry weight, root mass ratio, nodule dry weight, total leaf area, and leaf mass area in three *Cicer* species grown under low phosphorus.
**Fig. S2** Shoot phosphorus (P) concentration, root P concentration, shoot P content, root P content, physiological P‐use efficiency, P concentration ([P]) in the youngest fully expanded leaves for photosynthesis measurement, leaf nitrogen (N) concentration, and leaf N : P ratio in three *Cicer* species grown under low phosphorus.
**Fig. S3** Area‐based and mass‐based leaf photosynthesis rates, stomatal conductance, intercellular CO_2_ concentration (Ci), water‐use efficiency, photosynthetic phosphorus (P)‐use efficiency, and photosynthetic N‐use efficiency in three *Cicer* species grown under low phosphorus.
Please note: Wiley is not responsible for the content or functionality of any Supporting Information supplied by the authors. Any queries (other than missing material) should be directed to the *New Phytologist* Central Office.

## Data Availability

The data supporting the findings of this study are available in the Supporting Information of this article (Dataset S1, which includes all data for Figs 1, 2, 3, 4, 5, 6 and S1–S3).
