# Supplementary material for: Wild Cicer species exhibit superior leaf photosynthetic phosphorus‐ and water‐use efficiencies compared with cultivated chickpea under low‐phosphorus conditions
Source: New Phytol. 2025 May 5;247(1):144–59. doi: 10.1111/nph.70185 (PMC12138168; doi:10.1111/nph.70185)
Supplement: Supplementary file 2 — Fig. S1 Plant height, branch number, shoot dry weight, root dry weight, root mass ratio, nodule dry weight, total leaf area, and leaf mass area in three Cicer species grown under low phosphorus. Fig. S2 Shoot phosphorus (P) concentration, root P concentration, shoot P content, root P content, physiological P‐use efficiency, P concentration ([P]) in the youngest fully expanded leaves for photosynthesis measurement, leaf nitrogen (N) concentration, and leaf N : P ratio in three Cicer species grown under low phosphorus. Fig. S3 Area‐based and mass‐based leaf photosynthesis rates, stomatal conductance, intercellular CO2 concentration (Ci), water‐use efficiency, photosynthetic phosphorus (P)‐use efficiency, and photosynthetic N‐use efficiency in three Cicer species grown under low phosphorus. Please note: Wiley is not responsible for the content or functionality of any Supporting Information supplied by the authors. Any queries (other than missing material) should be directed to the New Phytologist Central Office. [file NPH-247-144-s001.docx]

***Supplementary Figure captions and Figures.***

**Wild *Cicer* species exhibit superior leaf photosynthetic phosphorus- and water-use efficiencies compared with cultivated chickpea under low-phosphorus conditions**

**Jiayin Pang**^*^, Simiao Li, Ulrike Mathesius, Jens Berger, Weina Zhang, Komal D Sawant, Rajeev K. Varshney, Kadambot HM Siddique, Hans Lambers

Article acceptance date: 11 April 2025

*Corresponding author. Email: Jiayin.Pang@uwa.edu.au

**Supplementary figure captions**

**Fig. S1.** (a) Plant height, (b) branch number, (c) shoot dry weight, (d) root dry weight, (e) root mass ratio, (f) nodule dry weight, (g) total leaf area, and (h) leaf mass area in three *Cicer* species—54 *C. reticulatum*, 15 *C. echinospermum* and seven *C. arietinum* accessions—grown under low−P conditions for nine weeks in a temperature-controlled glasshouse. Data are means ± s.e. (n=4). Vertical blue bars represent LSD_0.05_ among accessions.

**Fig. S2.** (a) Shoot phosphorus (P) concentration, (b) root P concentration, (c) shoot P content, (d) root P content, (e) physiological P-use efficiency, (f) P concentration ([P]) in the youngest fully-expanded leaves for photosynthesis measurement, (g) leaf nitrogen (N) concentration, and (h) leaf N:P ratio in three *Cicer* species —54 *C. reticulatum*, 15 *C. echinospermum* and seven *C. arietinum* accessions—grown under low−P conditions for nine weeks in a temperature-controlled glasshouse. Data are means ± s.e. (n=4). Vertical blue bars represent LSD_0.05_ between accessions.

**Fig. S3.** (a) Area-based and (b) mass-based leaf photosynthesis rates, (c) stomatal conductance, (d) intercellular CO_2_ concentration (Ci), (e) water-use efficiency, (f) photosynthetic phosphorus (P)-use efficiency, and (g) photosynthetic N-use efficiency in three *Cicer* species—54 *C. reticulatum*, 15 *C. echinospermum* and seven *C. arietinum* accessions—grown under low−P conditions for nine weeks in a temperature-controlled glasshouse. Data are means ± s.e. (n=4). Vertical blue bars represent LSD_0.05_ between accessions.

Supplementary Figure 1

Supplementary Figure 1 - continued

Supplementary Figure 2

Supplementary Figure 2 - continued

Supplementary Figure 3

Supplementary Figure 3 - continued
